# A Complementary Union of SARS-CoV2 Natural and Vaccine Induced Immune Responses

**DOI:** 10.3389/fimmu.2022.914167

**Published:** 2022-07-13

**Authors:** Joseph Torresi, Melissa A. Edeling, Terry Nolan, Dale I. Godfrey

**Affiliations:** ^1^ Department of Microbiology and Immunology, The Peter Doherty Institute for Infection and Immunity, University of Melbourne, Parkville, VIC, Australia; ^2^ Department of Infectious Diseases, The Peter Doherty Institute for Infection and Immunity, University of Melbourne, Parkville, VIC, Australia; ^3^ Murdoch Children’s Research Institute, Parkville, VIC, Australia

**Keywords:** COVID-19, neutralizing antibody, SARS-CoV-2, omicron, vaccines, immune imprinting

## Abstract

Our understanding of the immune responses that follow SARS-CoV-2 infection and vaccination has progressed considerably since the COVID-19 pandemic was first declared on the 11^th^ of March in 2020. Recovery from infection is associated with the development of protective immune responses, although over time these become less effective against new emerging SARS-CoV-2 variants. Consequently, reinfection with SARS-CoV-2 variants is not infrequent and has contributed to the ongoing pandemic. COVID-19 vaccines have had a tremendous impact on reducing infection and particularly the number of deaths associated with SARS-CoV-2 infection. However, waning of vaccine induced immunity plus the emergence of new variants has necessitated the use of boosters to maintain the benefits of vaccination in reducing COVID-19 associated deaths. Boosting is also beneficial for individuals who have recovered from COVID-19 and developed natural immunity, also enhancing responses immune responses to SARS-CoV-2 variants. This review summarizes our understanding of the immune responses that follow SARS-CoV-2 infection and vaccination, the risks of reinfection with emerging variants and the very important protective role vaccine boosting plays in both vaccinated and previously infected individuals.

## Introduction

COVID-19 has produced the greatest health challenge in more than 100 years causing over 547 million infections and 6.3 million deaths worldwide, fuelled by the emergence of SARS-CoV-2 variants which have become better adapted at infecting humans, overcoming prior immunity, and in some cases, resulting in more severe disease and a higher mortality (1–3). The current COVID-19 vaccines have proven to be highly effective and the impact of vaccines in reducing infections and disease severity has been substantial (4–8). Several important questions have arisen regarding the ability of vaccines to deliver long-term protection and whether individuals who have recovered from COVID-19 infection develop protective immune responses that are equivalent or complementary to vaccine induced immunity.

Neutralising antibody (NAb) responses following infection and vaccination play a central role in protection against SARS-CoV-2 infection. High affinity NAb mature over time and are stronger and more durable in symptomatic compared to asymptomatic individuals (9, 10). Some NAb also cross-neutralise SARS-CoV-1 (11, 12), responsible for the 2003/2004 SARS epidemic, suggesting that vaccines producing NAb responses to SARS-CoV-2 may potentially result in cross-protective immunity. Individuals infected with SARS-CoV-2 also develop virus-specific memory CD4+, cytotoxic T and B cells (13, 14). The importance of T cell immunity in both infection and after vaccination has recently been comprehensively reviewed (15). SARS-CoV-2 infection also results in polyfunctional memory T cell responses that persist for at least 12 months following infection. Severe acute infection is associated with higher frequencies of SARS-CoV-2 specific CD4+ T cell responses at 12 months (16). What is also apparent is that infection with SARS-CoV-2 elicits memory lymphocytes that persist and display the functional hallmarks associated with protective immunity.

The challenge emerging variants, such as Omicron, will present particularly for vaccine escape also creates many questions for current preventative and vaccination strategies. This review summarises the evidence for the development of protective immunity following recovery from SARS-CoV-2 infection and the role for booster vaccination in preventing infection with emerging variants (17). The nature of immune responses to both SARS-CoV-2 infection and vaccination in immunocompromised persons is a very important and complex topic that extends beyond the scope of this review and will not be discussed here.

## The Challenge of Omicron

The emergence of the Omicron variant is presenting new challenges for the control of the COVID pandemic and for vaccine development. Omicron was first detected in South Africa in November of 2021 (18) and has now spread globally, replacing Delta as the dominant global variant of concern (VOC) over a relatively short period of time (19) (20). Since its initial detection, disturbingly, Omicron has evolved into at least six into four sub-lineages – BA.1, BA.1.1, BA.2, BA.3, BA.4 and BA.5 (21) (22) with Omicron BA.1 and BA.2 producing large epidemics in many countries and BA.4 and BA.5 beginning to displace earlier omicron variants as the dominant sub-lineage. The transmissibility and severity of infection with Omicron BA.2 is similar to BA.1 (23) (24), although BA.2 is better able to escape NAb (25) (26) and reinfect individuals who have recovered from infection with Omicron BA.1 (27). At this stage, it is unclear if BA.4 and BA.5 variants lead to altered disease severity, but as they are rapidly overtaking other variants in several countries, they appear to be more transmissible.

**Figure 1:**
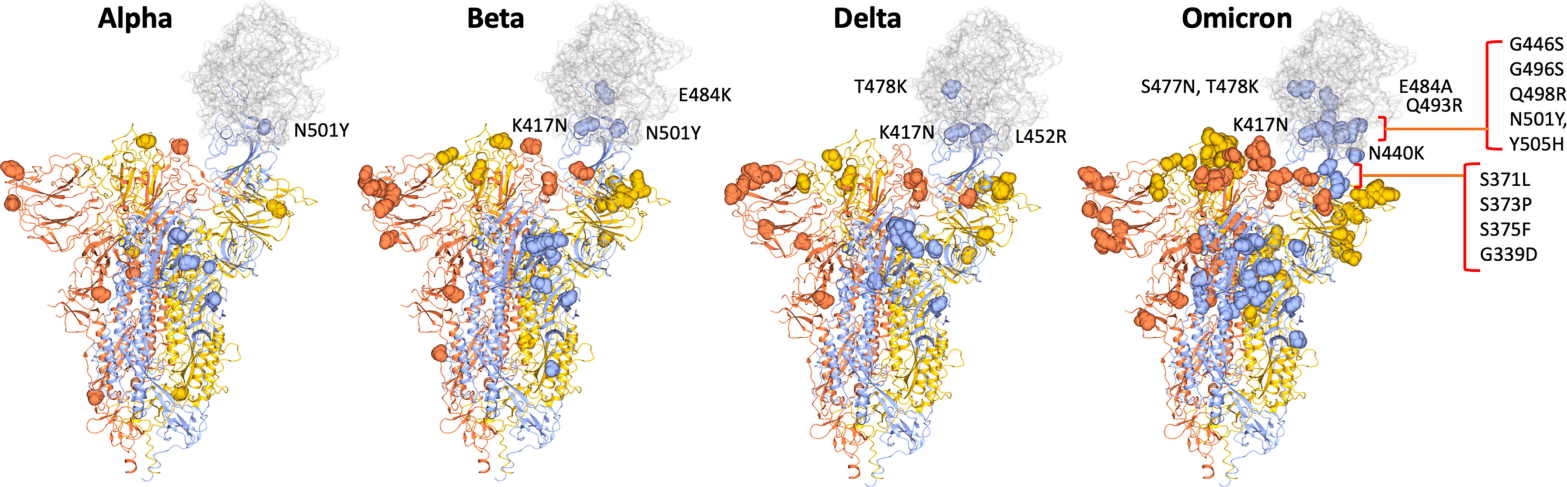
SARS-CoV2 spike trimer showing two RBDs in the closed conformation and one in the open conformation bound to ACE2 (grey) (PDB: 7DF4) (28). The position of changes in spike of the Alpha VOC through to Beta, Delta and finally the Omicron VOC are shown as yellow or orange spheres in the two spike monomers with closed RBDs and as blue spheres in spike monomer with open RBD, highlighting the proximity of mutations in relation to the binding site with ACE2. Mutations in RBD are labelled.

**Figure 2:**
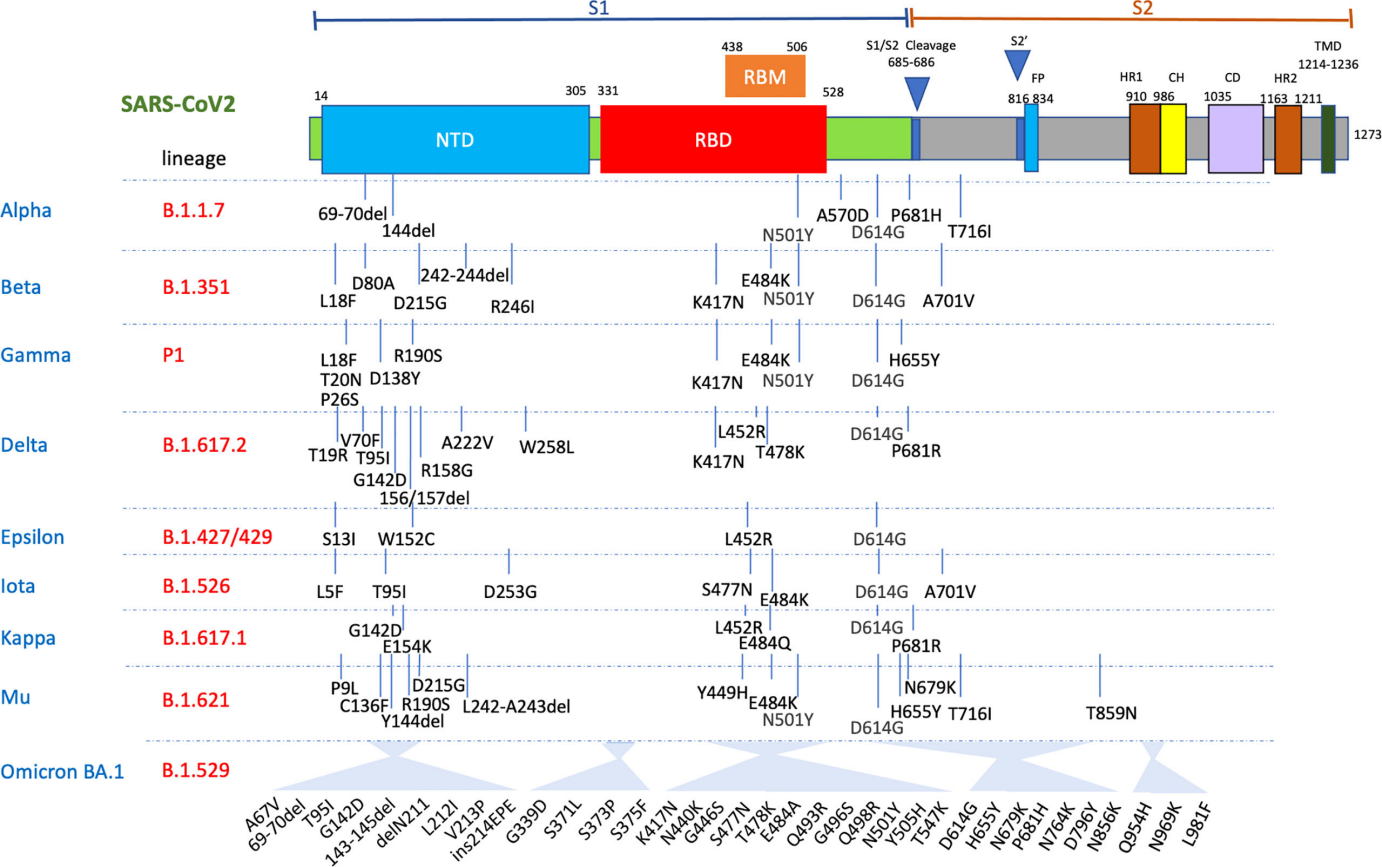
Mutations in the viral spike identified in Alpha, Beta, Delta, Gamma, Mu and Omicron BA.1 SARS-CoV-2 variants showing the significant clusters of changes in both the N-terminal domain (NTD) and RBD. FP, fusion peptide; HR1, heptad repeat 1; CH, central helix; CD, connector domain; HR2, heptad repeat 2; TMD, transmembrane domains.

Omicron (BA.1) carries 32 changes in the spike protein, with 15 in the receptor-binding domain (RBD) (responsible for attaching the virus to the ACE-2 receptor) alone (**Figures 1** and **2**). Several changes identified in the RBD of Omicron have previously been reported with the Alpha, Beta, Gamma and Delta variants (**Figures 1** and **2**) and are associated with reduced antibody neutralisation. The selection of variants like Alpha, Delta and Omicron with changes in the SARS-CoV-2 spike protein highlights that these changes are predominantly focused to the regions of the RBD and the upstream NTD, with Omicron possessing a greater number of changes within the RBD than previous variants (**Figure 1**) (28). Further changes are detected in the RBD of BA.4 and BA.5 variants, including changes that were previously associated with increased transmissibility of the Delta variant. These changes provide Omicron with a greater ability to escape vaccine-induced NAb with a resultant greater risk of reinfection. Although at the population level, Omicron appears to be associated with less severe infection, this likely reflects the greater ability for Omicron to circulate in immune and previously vaccinated populations compared to other VOC (23). Indeed, a recent study of over 500000 people in the UK (29) showed that for any given level of vaccination, Omicron resulted in a higher risk of hospitalisation compared to Delta.

A recent report from South Africa has shown that the risk of reinfection with Omicron is 2.39-fold higher than with Beta and Delta SARS-CoV2 (30). Also, a report from the Imperial College of London has shown that the risk of reinfection is as high as 5.4 relative to Delta (20). The doubling time of Omicron has also been reported to range from 2 to 3.6 days, ensuring that this VOC will spread rapidly through an immune population (20, 31).

## Naturally Induced SARS-CoV-2 Immunity Is Protective Against Reinfection

**Figure 3:**
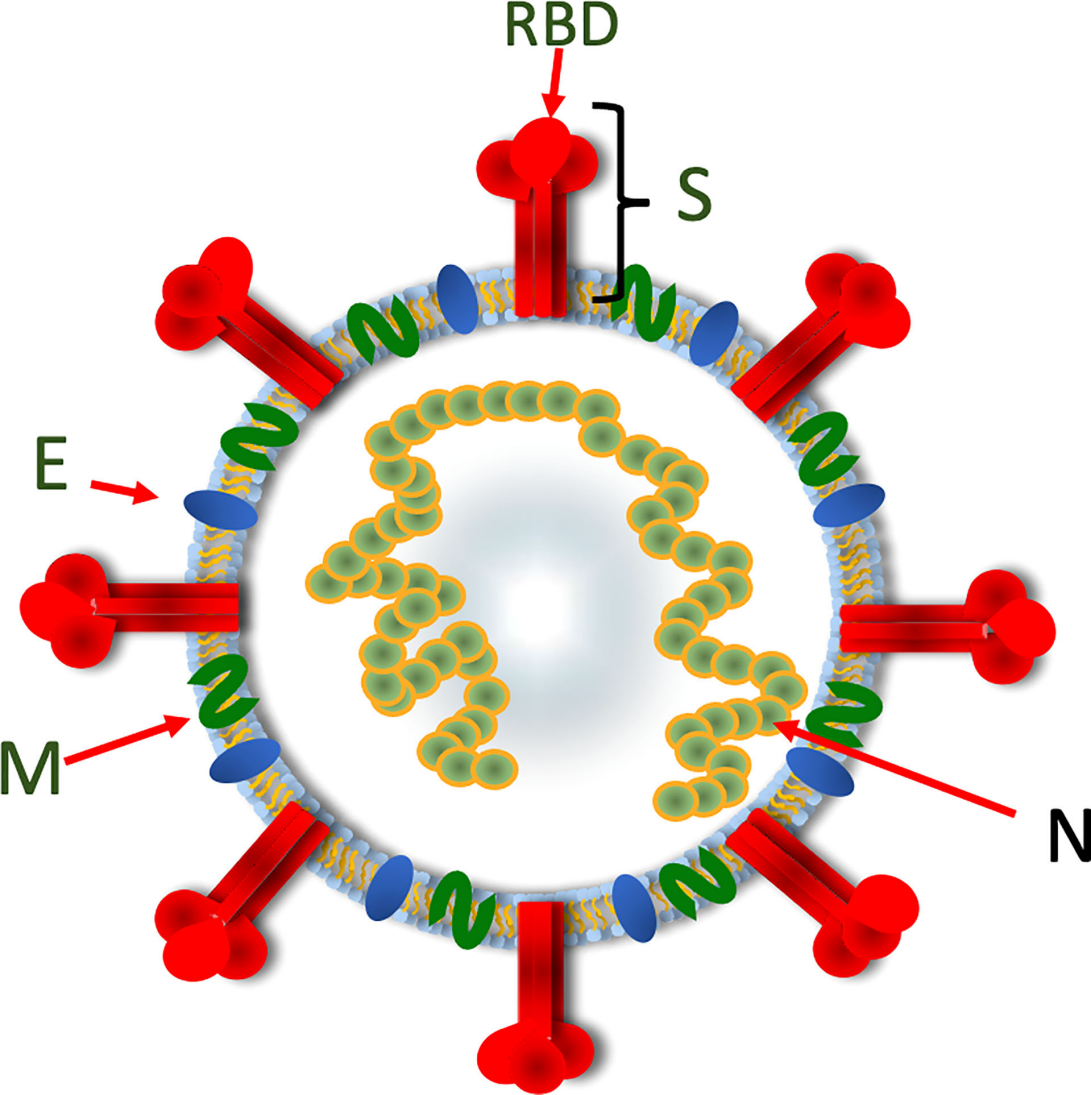
**A** diagrammatic representation of SARS-CoV-2 showing the organisation of the viral structural proteins. The spike protein assembles into a trimeric structure with the RBD located at the top of the trimer. The spike trimer is embedded in a lipid bilayer containing the membrane and envelope proteins. The nucleoprotein binds the viral RNA and also serves to stabilize the base of the spike trimer. Spike, S; Receptor-binding domain, RBD; Membrane, M; Envelope, E; Nucleoprotein, N.

**Figure 4:**
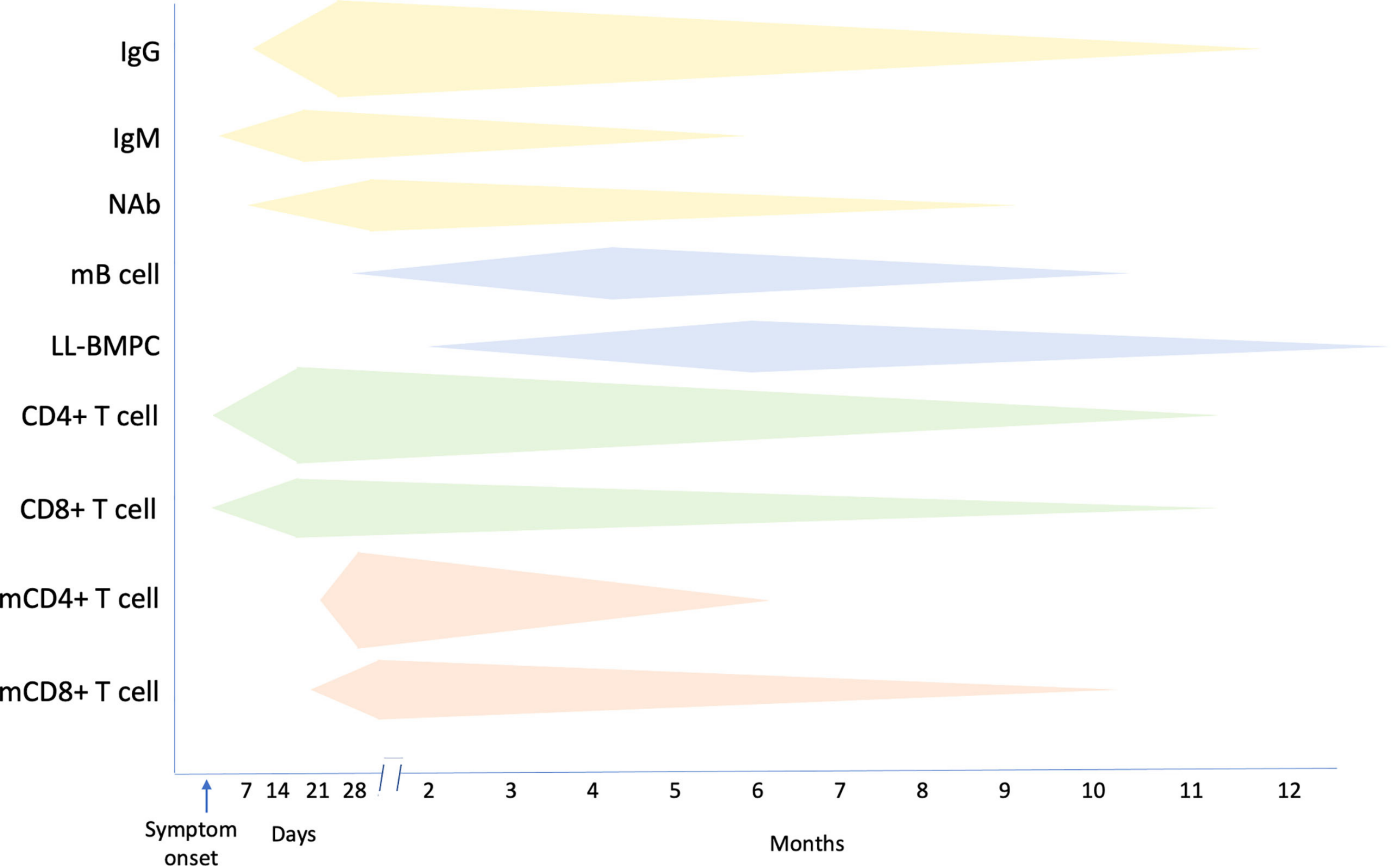
Summary of the kinetics of immune responses following SARS-CoV-2 infection. mB cell, memory B cell; LL-BMPC, long lived bone marrow plasma cell; mCD4+ T cell, memory CD4+ T cell; mCD8+ T cell, memory CD8+ T cell. Increasing magnitude of responses is represented by a greater width of respective bands.

Individuals who have recovered from COVID-19 infection develop protective immune responses that substantially reduce the likelihood of reinfection (**Figure 4**). Important protective immune responses develop to the major structural proteins of the virus, including spike (S), membrane (M), nucleoprotein (N) and envelope (E) (**Figure 3**). Following infection, patients develop antibody responses within the first 1 to 2 weeks that peak at weeks 3 to 4 (32, 33) and these remain detectable, albeit with a gradual decline, in the first 12 months after seroconversion (34). IgG responses appear to be the most durable, whereas, IgM and IgA titres wane more quickly by 6 months post infection (34–36). Potent NAb to the RBD and N-terminal domain (NTD) have been identified in convalescent sera (37, 38). NAb titres remain detectable for several months, with some studies suggesting they are generally stable over the first 6 months post infection (39, 40), while others suggest that anti-RBD antibodies wane over 4 months (41). In a study investigating the kinetics of antibody responses, 90% percentage of individuals were still seropositive for SARS-CoV-2 NAb 6 to 8 months after symptom onset (34).

Infection with SARS-CoV-2 elicits NAb responses that are most potent against variants carrying similar spike mutations and that are present in the immunising infection. By using pseudoviruses representing the spike proteins of different variants, it has been shown that Delta (B.1.617.2) SARS-CoV-2 is more strongly neutralised by convalescent serum from individuals who have had prior exposure to Delta or to Epsilon (B.1.429) or B.1.1.519, which have separate spike mutations that overlap with the spike mutations present in Delta (42). The strongest neutralisation was produced with serum elicited by homologous Delta exposure. In addition, exposure to SARS-CoV-2 antigens from infection together with vaccination plus a booster, results in broader *in vitro* neutralisation across different VOC, including Omicron (42) (**Figure 4**).

Strong and persistent memory B cells to RBD and spike protein and memory CD4+ and CD8+ T cell responses to membrane, nucleoprotein and spike proteins develop in most patients with COVID-19 (34, 43) and over a similar period, memory B cells increase while memory CD4+ and CD8+ T cells remain stable (34, 44, 45). While as expected, serum antibodies gradually wane following infection, nonetheless, NAb to RBD, and RBD-specific memory B cells, can persist for up to 12 months after infection (46). In one study, SARS-CoV-2-S-specific memory B cells could be identified in the blood of convalescent patients as early as one month and were maintained for up to 7 months after symptom onset (47, 48). The majority of SARS-CoV-2 infected individuals also develop class-switched memory B cells to several viral proteins including S and RBD, and durable SARS-CoV-2-specific B cell responses develop after mild or severe disease (44). Long-lived S-specific bone marrow plasma cells (BMPC) and memory cells that provide a persistent source of protective antibodies against SARS-CoV-2 can also be detected for up to 12 months (47, 48). In one study, investigators obtained bone marrow aspirates from convalescent individuals at 7 and 11 months after infection and enriched BMPC before quantifying the frequencies of SARS-CoV-2 S-specific IgG and IgA by Elispot. Both IgG- and IgA-secreting S-specific BMPCs were detected in 15/19 and 9/19 individuals respectively. None of these convalescent patients had S-specific antibody-secreting cells in blood at the same time point, indicating that the BMPC responses only represented resident bone marrow cells. The IgG- and IgA-secreting S-specific BMPCs were also present in the second sample time 4 months later, indicating that the BMPC populations were stable (47, 48). Furthermore, similar frequencies of RBD- and S-specific memory B cells from individuals who have recovered from infection with ancestral SARS-CoV-2 strain are able to target RBDs of both Alpha and Beta variants (48) (**Figure 4**).

Polyfunctional CD8+ and CD4+ T cell responses to the viral structural proteins can be detected in most patients who have recovered from SARS-CoV-2 infection (14, 49–52). Circulating SARS-CoV-2-specific CD8+ and CD4+ T cells can be identified in blood samples collected 20 to 35 days after onset of symptoms in 70% and 100% of COVID-19 convalescent patients, respectively (34, 53). However, the half-life of SARS-CoV-2 specific CD4+ and CD8+ T cells is only 3 to 5 months (34). Most convalescent individuals develop robust CD4+ T cell responses to spike (S)-derived peptides that correlate with the magnitude of the anti-SARS-CoV-2 IgG and IgA antibody responses (53). These include CD4+ T cell responses to the RBD, observed in 95% of individuals (54) and also responses to peptides from other viral structural and non-structural proteins, although the structural proteins S, membrane (M) and nucleoprotein (N) specific T cell responses are most strongly correlated with recovery and are the most prominent targets of SARS-CoV-2-specific CD4+ T cells (13, 14, 55–58) (**Figure 4**).

Memory CD4+T and CD8+ T cell responses are present in over 90% of recovered individuals in the first month after infection and remain constant over the first 8 months (34, 49) (50). The half-life of SARS-CoV-2 S-specific memory CD8+ T cells has been reported to be approximately 185 days (34). SARS-CoV-2-specific memory CD4+ T cell responses are more robust than memory CD8+ T cell responses, with over 90% of individuals having detectable circulating SARS-CoV-2-specific CD4+ T cells and of these 40% having more than 1% SARS-CoV-2-specific CD4+ T cells one month after symptom onset. At 6 months after symptom onset, 92% will still have detectable SARS-CoV-2-specific memory CD4+ T cells (34) (**Figure 4**).

In humans, the RBD is also highly immunogenic for T cells, comprising a cluster of peptides recognised by 94% of individuals (52). Patients recovering from severe COVID-19 infection have higher levels of S-specific memory B cells compared to patients with mild infection (34, 43). In recovered individuals, the magnitude of polyfunctional memory CD4 T cell responses to M protein is greater than responses to both N and S proteins (43). The frequency of M-specific CD4+ T cells also correlates with frequency and BCR avidity of S1-specific memory B cells (43). In the case of infection with the original SARS-CoV-1 virus, memory CD8+ T cells responses have been detected 17 years after recovery. The development of long-lasting memory CD8+ and CD4+ T cells may also be possible following COVID-19 infection (49, 52). Following mild infection with SARS-CoV-2, specific CD8+ T cells, particularly to the nucleoprotein, undergo continuous differentiation to form long-lived memory T cells and are associated with protection against severe disease (45, 51, 59). Individuals who have recovered from SARS-CoV-2 develop durable circulating RBD and S-specific circulating T follicular helper (cTFH) cells that correlate with the development of memory B cells and NAb and durable CD4+ and CD8+ T cell responses that form an important defence against reinfection (13, 34, 60). Convalescent-phase SARS-CoV-2-specific CD8+ T cell responses are also skewed towards a differentiated memory phenotype in contrast to acute phase T cells that display an activated cytotoxic phenotype (13).

Before the appearance of Omicron, several studies from the preceding time periods had shown that reinfection of SARS-CoV-2 previously infected individuals was relatively infrequent (61). Pre-existing SARS-CoV-2 antibody positivity, from previous infection or vaccination, has been associated with up to 90% lower likelihood over a 90-day period of becoming infected with SARS-CoV-2 compared to antibody-negative individuals (62). A US study of healthcare workers (HCW) in Cleveland found no cases of reinfection over a 5-month period among individuals who had previous SARS-CoV-2 infection, half of whom had also not received a COVID vaccine (63). A further study from Denmark examining reinfection rates over a 6-month interval separating two COVID-19 surges found that 80.5% of individuals aged under 65 years were protected against reinfection compared to 50% of those over 65 years (64). In Austria, reinfection was reported in 0.27% of individuals with past SARS-CoV-2 infection compared to 2.85% of the general population experiencing a first infection, equating to a 90% reduction in the risk of becoming reinfected (65). A study of US marine corp recruits reported an 82% reduction in SARS-CoV2 reinfection in seropositive individuals. In addition, reinfected individuals had higher PCR Ct values and 10-fold lower viral loads than seronegative individuals (66).

A large multicentre, prospective cohort study of UK HCW conducted between June 2020 and January 2021 also reported a low rate of reinfection compared to primary infection (67). Within this cohort, the number of cases, or incidence density for reinfection, was 7.6 per 100,000 person-days in the positive cohort compared with 57.3 primary infections per 100,000 person-days in the negative cohort. HCWs with a previous history of infection with SARS-CoV-2 had an 84% lower risk of infection with a protective effect lasting at least 7 months from the time of primary infection (67).

Furthermore, past infection has been shown to result in a level of protective immunity that is comparable to that achieved with two doses of the BNT162b2 mRNA (Pfizer–BioNTech) vaccine (68). In this study, investigators found that the BNT162b2 mRNA vaccine had an overall efficacy of 92.8% for infection, 94% for hospitalisation, severe illness and death. In comparison, the level of protection for individuals with prior infection was found to be 94.8% for infection, 94% for hospitalisation and 96.4% for severe illness (68).

Previous SARS-CoV2 infection also provides a greater level of protection against variants following a booster dose of vaccine. Sera from individuals who have recovered from SARS-CoV-2 are able to neutralise the ancestral strain but the neutralising activity towards Beta SARS-CoV-2 (a known neutralising escape variant) is 10-fold lower. In contrast, after a single booster of BNT162b2 mRNA or mRNA1273 vaccine, NAb titres against the Beta variant 14 days after the booster are increased by up to 1000-fold (69).

## Benefits of Vaccination After Recovery From SARS-CoV-2 Infection

Natural immunity after COVID-19 infections provides partial protection against reinfection, although this has now been shown to be less effective against Omicron. However, what has become clear is that vaccinating individuals who have recovered from COVID-19 provides an additional benefit by boosting protective immunity against variants, including Omicron. Vaccine effectiveness against Omicron remains higher than 90% in previously infected individuals who were subsequently vaccinated (70).

A recent study comparing the immune responses to mRNA vaccines in both SARS-CoV-2 naïve and recovered individuals demonstrated that SARS-CoV-2 specific CD4+ T cell responses correlated with SARS-CoV-2 IgG responses. However, some studies have suggested that naïve individuals required two doses of an mRNA vaccine to achieve a high level of protective immunity while COVID-19 recovered individuals achieved peak immunity after one dose of vaccine (71). Individuals with past SARS-CoV-2 infection develop high level durable antibody responses following vaccination with mRNA vaccines compared to individuals without past infection, with an absolute difference of 2.6-fold 6 months after vaccination (72). RBD-specific antibody titres also increase more than 30-fold and NAb almost 50-fold in vaccinated compared to unvaccinated individuals with previous COVID-19 infection. Vaccination of individuals who have recovered from COVID-19 has been shown to significantly increase NAb titres against Alpha, Beta, Iota and Gamma variants and produce an almost 9-fold increase in the number of circulating RBD-specific memory B cells (46). Vaccination of individuals with past SARS-CoV-2 infection also results in somatic hypermutation and affinity maturation of antibodies, a greater and more persistent memory B cell clonal expansion and the development of antibodies that retain binding activity against RBD variants that could potentially be protective against SARS-CoV-2 variants (46).

In SARS-CoV-2 seronegative individuals, a single dose of an mRNA vaccine results in relatively low antibody titres in the first 12 days after vaccination, with the majority achieving high antibody titres after 21-27 days and a second dose of vaccine producing more than a doubling of antibody levels. In contrast, SARS-CoV-2 seropositive individuals develop high antibody levels within a week after a single dose of vaccine, with antibody titres 10 to 45-fold higher than seronegative individuals at the same time points after the first vaccine dose (73).

A study from Qatar reported that infection in previously uninfected individuals who were vaccinated with Pfizer BNT162b2 or Moderna mRNA-1273 vaccines was significantly more frequent than in individuals who received these vaccines following past SARS-CoV-2 infection. These investigators found that incidence of infection in BNT162b2 mRNA-vaccinated individuals was 0.15% in those with, versus 0.81% in those without, prior SARS-CoV2 infection over a 120-day follow-up period. In individuals who had received mRNA-1273 vaccine, the incidence of infection was 0.11% in those with and 0.35% in those without past SARS-CoV2 infection (74).

Before the emergence of Omicron, providing a second or booster dose of vaccine in previously infected individuals appeared to be of less benefit, as highlighted in studies comparing a single dose with two doses of the BNT162b2 mRNA vaccine (75, 76). Furthermore, in previously infected individuals, a second dose of BNT162b2 mRNA vaccine did not result in NAb levels that were higher than levels following one dose of vaccine (76). Robust cytotoxic CD8+ and CD4+ T cell responses together with antigen-specific antibody secreting cell responses can also be detected after a first vaccine dose that are not improved with a second vaccine dose (75). However, as new variants emerge, a single dose of a COVID-19 vaccine has been shown to be less capable of generating protective immune responses in SARS-CoV-2 previously infected individuals (77). This has been highlighted in a recent report from Israel showing that a fourth dose of COVID-19 mRNA vaccine, although producing an almost 10-fold rise in NAb, was only modestly effective at enhancing protection against infection compared to that of the third dose, with vaccine efficacies of 30% and 11% for BNT162b2 mRNA and mRNA1273 vaccines respectively. However, breakthrough infections were only mild, reinforcing the benefit of vaccination in protecting against severe COVID-19 disease (77).

## Vaccine Efficacy, Boosting and VOC

The SARS-CoV-2 virus has evolved into variants that are substantially more infectious and are able to evade NAb that are directed to the RBD and receptor-binding motif (RBM) of the S protein (78, 79). Following initial reports describing the D614G variant, several additional mutations in S1 and RBD proteins have been reported that enhance binding of viral RBD to human ACE-2 receptor, increasing virulence and transmissibility (80–82) (**Figure 2**). Over the past 2 years, successive waves of SARS-CoV-2 variants have completely replaced not only the ancestral wild-type SARS-CoV-2 virus, but also the previously dominant variants. The most prevalent circulating variants carry combinations of changes within the S1, not just RBD and have included Alpha (83), Beta (84), Epsilon (1, 85), Delta (86) and most recently Omicron and its sublineages (18) which are associated with greater transmission and disease severity (1, 85, 87–89) (**Figure 3**).

Most COVID-19 vaccines have relied on the delivery of the spike (S) or the RBD components to produce S-specific humoral immune responses, including NAb responses (90–94). The current vaccines produce humoral immune responses not only to the ancestral strain of SARS-CoV-2 but also to VOC (46). Whilst current vaccines have been effective in producing targeted antibodies that neutralise SARS-CoV-2, it is becoming increasingly evident that they are less effective in neutralising and preventing infection with emerging SARS-CoV-2 VOCs (1, 95–99). This may be especially problematic with the Omicron variant (18) (**Figure 3**).

Importantly, pre-existing immunity will also determine the relative ability of next generation vaccines to build-on current vaccine immunity and to broadly boost immune responses including memory B cells as well as CD4+ and CD8+ T cell responses. A study from Israel has shown that previous SARS-CoV-2 infection provided a significant level of protection against reinfection including with the SARS-CoV-2 Delta variant (100). These investigators demonstrated that SARS-CoV-2 naïve individuals vaccinated with BNT162b2 had a 6-13-fold increased risk, depending on time of prior exposure, for breakthrough infection with Delta compared to previously infected individuals (100). Conversely, however, a recent US study of COVID-19 hospitalisations among adults aged ≥18 years who had previous infection or vaccination 90–179 days earlier, reported a more than a 5-fold higher likelihood of infection amongst unvaccinated, previously infected individuals compared to fully vaccinated recipients of two doses BNT162b2 mRNA or mRNA-1273 vaccine and who had not previously been infected with SARS-CoV-2 (101).

The benefit of booster doses has also been shown in recipients of the BNT162b2 mRNA vaccine. In naïve individuals, two doses of BNT162b2 mRNA vaccine produces a strong NAb response against ancestral SARS-CoV-2, but less so for the Beta and Delta variants (102). By administering a third dose of 30 µg of vaccine 7.9 to 8.8 months after the second dose in individuals aged 18 to 85 years, by 1 month after dose 3, NAb titres against wild type SARS-CoV-2 increased more than 5 times in individuals aged 18 to 64 years and more than 7 times in those aged 65 to 85 years. More significantly, NAb titres against the Beta variant increased more than 15 times in the 18 to 64 and more than 20 times in the 65 to 85 year old age group (102).

Similarly, in naïve individuals, two doses of vaccine were necessary to achieve an optimal frequency of SARS-CoV-2 S and RBD-specific memory B cells seven days after a second dose of BNT162b2 mRNA vaccine, with memory B cells persisting up to 6 months post vaccination (103–105). Interestingly, individuals who have recovered from SARS-CoV-1 infection develop high level cross-clade NAb to SARS-CoV-2 variants 21 to 62 days after receiving a first dose of BNT162b2 mRNA vaccine develop (106). The importance of boosting is further highlighted in a recent report showing that a third dose of the BNT162b2 mRNA vaccine is associated with an 86% reduction in the risk of reinfection 28 to 65 days following receipt of the booster and up to a 97% reduction in hospitalisation in individuals who previously had two doses of vaccine (107). The effectiveness of the third dose also increased over time following receipt of the booster, with only a 12% reduction in the risk of infection in the first 7 days, 58% reduction 7 to 13 days and rising to 85% in days 14 to 20 and beyond (107).

In studies from Israel, the emergence of the Delta variant resulted in a resurgence of COVID-19 in a population that had prior relatively high vaccination rates. However, the introduction of a booster dose of BNT162b2 mRNA vaccine at least 5 months after a second dose of vaccine resulted in a 90% reduction in deaths due to COVID-19, again highlighting the importance of booster vaccination against SARS-CoV-2 variants (108, 109).

Finally, another recent study has shown that infection plus two vaccine doses (3 weeks apart) or three doses of BNT162b2 mRNA vaccine (with the third dose given 9 months after the primary series) produces a greater capacity in a live virus neutralisation assay to neutralise non-homologous SARS-CoV-2 variants including a 42-fold increase in naïve and 14-fold in convalescent individuals against Omicron (110). In convalescent individuals, a progressive increase in antibody titres and avidity occurs in the first 7 months after 2 doses of vaccine that does not increase further after a third dose (110). Although booster doses restore NAb titres against Omicron, a recent study from Israel has shown that a fourth dose of either BNT162b2 mRNA or mRNA1273 vaccine administered 4 months after a third vaccine dose produced a 10 fold increase in NAb titres that were only marginally higher than those after the third dose and a vaccine efficacy of only 30% and 11% higher than individuals who had received three doses of these vaccines respectively (77).

T cell responses also contribute to protection against VOCs. In a study investigating longitudinal T cell responses in naïve and convalescent individuals before and two weeks after the first and second doses of vaccine, S-specific T cell responses elicited by vaccination were detected to similar levels against the ancestral, Alpha and Beta variants. In infection naïve individuals, S-specific CD4+ T cell responses were primed with the first dose of vaccine and further boosted with the second dose. In contrast, in convalescent individuals, a second dose of vaccine did not increase CD4+ T cell responses (111). In addition, spike-specific CD4+ and CD8+ T cells elicited by vaccination respond in the same manner to epitopes from ancestral virus as they do to epitopes from both the Alpha and Beta variants (111). The mutations present in SARS-CoV-2 variants (including Alpha, Beta, and Gamma) have also been shown to have little impact on sequences of both CD4+ and CD8+ T cell epitopes in both SARS-CoV-2 previously infected individuals and vaccine recipients (112). Convalescent individuals have similar strong CD4+ and CD8+ T cell responses to peptide pools representing the S proteins of both the ancestral and variant SARS-CoV-2 viruses CD4+ and CD8+ T cell epitopes in both SARS-CoV-2 exposed individuals and vaccine recipients (112). More recently, it has also been shown that memory CD4+ and CD8+ T cells produced 2 weeks after mRNA-1273 and BNT162b2 and 6 weeks after Ad26.COV2.S vaccines are able to cross-recognize variants Alpha, Beta, Delta, Gamma and Omicron and these responses are preserved for at least 6 months after the last vaccination. In contrast, memory B cells responses to variants are reduced, particularly to Omicron (113). These findings help to explain why current SARS-CoV-2 vaccines are protective against hospitalisation and the development of severe disease following infection with SARS-CoV-2 variants.

## Severity of Omicron and Protection by Vaccination

Several studies have suggested that infection with Omicron is less likely to result in severe infection (17, 19, 20, 114). In South Africa, infection with Omicron was associated with an 80% lower risk of hospitalization compared to other VOC and a 70% lower risk compared to infection with Delta SARS-CoV-2 (19). However, the risk of severe disease once hospitalised was similar for Omicron compared to other VOC (19). In a subsequent study from Tshwane in the Gauteng Province, South Africa, the proportion of deaths related to Omicron infection was 4.3% compared to 21.3% for previous variants. Admissions to ICU were reduced from 4.3% to 1% and the length of hospital stay was reduced by over half (4 compared to 8.8 days). COVID-19 pneumonia was reported in one third of patients infected with Omicron, of which 72% of patients had mild to moderate disease. Finally, of patients admitted to hospital only 45% required supplemental oxygen compared to 99% in previous waves of COVID-19 (115). However, in another study where Omicron was compared to Delta in unvaccinated and age-matched individuals, the severity of Omicron was found to be close to that of Delta, with a hazard ratio of 0.75 (114). The key point appears to be that Omicron can be of similar severity to previous variants in naïve individuals, but because it tends to infect vaccinated and/or convalescent individuals, its severity appears to be lower than earlier variants.

Similarly in the UK, the risk of hospitalisation for individuals infected with Omicron is significantly reduced compared to Delta infection (17, 20). Hospital attendances were reduced by 25% while hospitalisation lasting more than one day was reduced by up to 45%. Furthermore, previous SARS-CoV-2 infection reduced hospitalisation by 50% while the risk of hospitalisation for one day or more was reduced by 69%. Previous infection with a homologous variant has also been found to be 90% protective against reinfection with Alpha, 86% against Beta, 92% against Delta but only 56% against Omicron. However, protection against developing severe or fatal COVID-19 was 69% for Alpha, 88% for Beta, 100% or Delta and 88% for Omicron (116).

Omicron escapes vaccine-induced NAb with studies showing that neutralisation of Omicron by vaccinee immune sera is reduced 20 to 85 fold (88, 89, 117, 118). In addition, in convalescent sera from patients who had been previously infected with Alpha, Beta, Gamma or Delta SARS-CoV-2 variants, neutralisation titres for Omicron are reduced 18-fold with Alpha, 22-fold with Beta, 12-fold with Gamma and 26-fold with Delta convalescent sera (89). Despite the reduction in Omicron neutralisation, boosting with the BNT162b2 mRNA vaccine resulted in a 34- (89) to 100-fold (119) increase in NAb titres against Omicron (89). Similarly, a 50µg booster dose of mRNA-1273 vaccine results in a 12-fold improvement in neutralisation of Omicron and a 3-fold improvement in neutralisation of Beta compared with D614G virus (120).

Vaccinated individuals who become infected with Omicron also develop broader protective immune responses against other SARS-CoV-2 variants and 10-fold higher NAb titres against Omicron than infected unvaccinated individuals (121, 122). A similar pattern has been observed with NAb against Delta, with Omicron infected vaccinated individuals developing higher titres of NAb against Delta compared to Omicron infected unvaccinated individuals (121). In individuals previously infected with Delta and subsequently infected with Omicron, NAb titres against Delta are 22-fold higher than NAb against Omicron (121). In contrast, unvaccinated individuals, and naïve mice, infected with omicron, fail to develop broad NAb responses against Delta (121, 122).

Vaccine effectiveness against infection with Omicron is also reduced (20, 123, 124). A report from the UK has shown that two doses of ChAdOx1 had little effectiveness against Omicron infection, despite remaining highly effective against severe disease. Vaccine effectiveness after two doses of BNT162b2 mRNA vaccine was as high as 88% in the first 2 to 9 weeks after vaccination but this fell to 37% from 15 weeks after the second dose of vaccine (123). However, vaccine effectiveness increased to 71% for ChAdOx1 and 75% for BNT162b2 mRNA primary course recipients two weeks after a BNT162b2 mRNA booster, indicating that 3 doses of vaccine can substantially boost protection against Omicron. For Delta, vaccine effectiveness was 42% at 25 weeks after two doses of ChAdOx1 vaccine, increasing to 94% two weeks after a BNT162b2 mRNA booster. For individuals who had a BNT162b2 mRNA primary course of vaccination, vaccine effectiveness was 64% 25 weeks after 2 doses of vaccine, increasing to 93% two weeks after a booster (123). In a follow-up study from South Africa, the efficacy of two doses of the BNT162b2 mRNA vaccine against hospitalisation was found to be 70% for Omicron compared to 93% for the Delta variant (125). A further study from Israel has shown that a third dose of the BNT162b2 mRNA vaccine results in a 93% reduction for hospital admission, 92% for severe disease and 81% for death, starting 7 days after receipt of the booster dose (124). Vaccine boosters therefore enhance protection against both Delta and Omicron, albeit with greater protection against Delta (126).

Omicron has emerged in a setting of populations that are already highly vaccinated and/or previously infected with SARS-CoV-2, indicating that the virus is continuing to evolve in a way that will enable it to escape immunity of current COVID-19 vaccines. While Omicron is often viewed as a milder infection, studies that have focused on Omicron infection in unvaccinated and uninfected individuals have suggested that the intrinsic severity of Omicron may not be much lower than Delta. Regardless, it is clear that the emergence of Omicron and its subvariants, and new VOC that will undoubtedly arise in the future, will continue to bring new challenges for the development of next generation vaccines.

## Immune Imprinting: A Partnership of Infection and Vaccine Induced Immunity

The breadth and specificity of antibody responses that develop following infection with SARS-CoV-2 variants may be substantially influenced by imprinting of immune responses to prior coronavirus infections and COVID-19 vaccination (127–129). Immune imprinting refers to the impact of an initial exposure to a virus or vaccine on shaping the specificity of subsequent immunological responses which are biased towards the original virus antigen exposure, potentially constraining the development of immune responses to new antigens associated with variant strains. The implications of immune imprinting are that the first variant of a virus that an individual is exposed to will limit the ability of that person to mount a response to antigenically distinct variants of that virus. With increasing age, a person will acquire antibodies to new strains of a virus while maintaining high levels of antibodies to the original virus variant. The advantage of imprinting is that it may provide protection against specific antigenic subtypes (130), although this may occur at the cost of developing strong protective immune responses against variants a person may encounter subsequently (131). Immune imprinting has been well described with influenza infection and vaccination (132) and more recently reported with SARS-CoV-2 infection (127–129). How this immune history may affect responses to vaccines or new viral variants in the setting of COVID-19 infection is not fully understood, although it has important implications for future vaccination approaches.

Previous studies of influenza infection and vaccination may provide some insight into what might be expected with COVID-19. Influenza antibodies bind strongly to an original viral strain against which these antibodies were initially elicited while binding poorly to variants that have drifted from the founding strain (133–136). However, if emerging variants remain antigenically related to the ancestral virus, then antibodies to new variants remain capable of protecting against future infections (135). Imprinted antibody responses may therefore be beneficial, providing protection during secondary exposures with drifted viral variants. However, imprinting by pandemic strains of influenza early in life might also increase susceptibility to antigenically distant strains during future pandemics (133–135). A similar phenomenon has been observed with influenza vaccines. The efficacy of influenza vaccines from year to year is impacted by the antigenic relatedness of past vaccines, the strains represented in current vaccines and circulating epidemic viral strains. Vaccine efficacy can be predicted to be high when the antigenic distance between all three strains is close but likely to be low if the antigenic distance between the vaccine and epidemic strains is distant (133, 134). Similar factors may well apply to current COVID-19 vaccines and emerging variants like Omicron BA.1, BA.2, BA.4 and BA.5. It is possible that a potential bias in immune response towards an earlier infecting variant or an ancestral COVID-19 vaccine could possibly impede or, alternatively, compliment an immune response to variant-specific vaccines. Repeated influenza vaccination of individuals previously infected with an antigenically distant virus earlier in life also results in a lower vaccine efficacy compared to individuals who have had past infection with a closely related virus (136).

A recent study investigating antibody responses following COVID-19 mRNA vaccines has shown that the breadth of antibody responses to viral variants is less after infection compared to vaccination, although the former improves over the ensuing months (128). Individuals who were only exposed to ancestral antigens through either infection or BNT162b2 mRNA vaccine exhibited a hierarchy of IgG responses to variant RBDs (Epsilon>Kappa>Delta>Gamma>Beta) relative to ancestral RBD. Infection with viral variants also elicits variant-specific antibodies. Prior mRNA vaccination with an ancestral vaccine followed by Alpha or Delta infection resulted in stronger antibody response towards the ancestral virus and decreased antibody responses to viral variant epitopes compared to unvaccinated individuals infected with these variant viruses. In contrast, antibody from individuals infected with Alpha or Delta variants and with no prior history of vaccination showed improved binding to Alpha or Delta variant RBDs compared to ancestral RBD (128). These findings suggest that antibody responses are determined in part by the imprinted responses to the initial infection, be it with ancestral or a variant virus. In another study of longitudinal immune responses in health care workers, investigators found that following exposure to three antigens through infection with Alpha plus two doses of an ancestral COVID-19 vaccine, heterologous neutralising antibody titres against Beta, Gamma and Delta variants plateaued while neutralising immunity to Alpha increased, suggesting imprinting of humoral immune responses towards the earlier variant (129).

In another study looking at the ability of immune sera from individuals vaccinated with two doses of the mRNA-1273 vaccine to neutralise the Omicron variant using a pseudovirus assay it was found that neutralisation titres were 35 times lower against Omicron compared to the D614G variant alone (118). However, a booster dose of the mRNA-1273 vaccine resulted in neutralisation titres against Omicron that were 20-times higher than one month after the second dose of vaccine (118). Boosting with a bivalent ancestral and Beta (mRNA-1273.211) or Beta and Delta (mRNA-1273.213) vaccine resulted in almost identical neutralisation titres for Omicron as individuals who received a booster with mRNA-1273 alone (118) (137). Interestingly, in a recent study that investigated protective immune responses against Omicron in macaques immunized with mRNA-1273 or an mRNA-Omicron vaccine (138), there was no increased response to Omicron in animals boosted with the Omicron vaccine. Macaques were immunized with two doses 4 weeks apart of mRNA-1273 vaccine and boosted at week 41 (9 month) with mRNA-1273 or mRNA-Omicron vaccine. By week 6 after primary immunization, a hierarchy of antibody responses was observed, with the strongest responses against ancestral virus followed by the more closely related Delta virus, then Beta and finally Omicron (138). At nine months after the second dose of mRNA-1273 vaccine, macaques were boosted with homologous mRNA-1273 or heterologous mRNA-Omicron vaccine. Both homologous and heterologous boosting restored titres of anti-spike antibody and NAb (measured by live virus neutralisation and pseudotype assays), cross reactive memory B and cTfh cells to the same levels and with the same hierarchy of response as observed at week 6 post-second dose. Furthermore, homologous boosting provided equivalent protection against challenge with Omicron virus as did heterologous boosting with mRNA-Omicron vaccine (138). Taken together, these studies leave open the question of whether variant-targeting vaccines can overcome imprinting and improve variant-specific immunity, and further investigation is clearly required in this area.

Whilst humoral immune responses may be impacted by imprinting of prior antigen exposures, memory T cell responses induced by COVID vaccines (including mRNA-1273, BNT162b2 mRNA, Ad26.COV2.S, and NVX-CoV2373) appear to remain preserved against several variants (113). In the 6-month period after vaccination, up to 90% of CD4+ and CD8+ T cell memory responses to spike megapeptide pools measured by Activation Induced Marker (AIM) assay are preserved against variants, including Alpha, Beta, Gamma and Delta. For Omicron, the conservation of memory CD4+ and CD8+ T cell recognition is up to 85% whilst NAb and memory B cell responses are reduced by more than 50% (113). The preservation of vaccine-induced cross-reactive T and B cell responses against SARS-CoV-2 variants is encouraging for the longer-term protective benefits of vaccination (15). Thus, while immune imprinting with one SARS-CoV-2 virus or exposure to vaccine representing an earlier viral variant may limit the development responses to new antigenic targets on new variants, the abundance of shared epitopes means that protective benefits are still afforded by current, and probably future, COVID-19 vaccines.

## Conclusion

Whether immunisation comes from prior infection with SARS-CoV-2, or from vaccination against SARS-CoV-2, or a combination thereof, the results are development of effective immune responses that protect against progression to severe disease and at least partial protection to SARS-CoV-2 VOC. However, natural and vaccine-induced immunity wanes with time, and new variants are evolving that can evade existing immunity. Consequently reinfection with SARS-CoV-2 variants is increasingly possible, indeed, likely. What is becoming clear is that boosting previously vaccinated or SARS-CoV-2-infected individuals substantially increases the level of protection against new circulating variants, including Omicron. Whilst there is debate around the role of imprinting of SARS-CoV-2 immune responses and how this might restrict the development of more broadly protective responses, it is also feasible that immune imprinting may offer opportunities to target epitopes that are shared between different SARS-CoV-2 variants, much as has been described with infection by antigenically similar influenza viruses. Next generation COVID vaccines will need to target conserved neutralising epitopes of the virus, in a similar manner to broadly cross-reactive antibodies that arose when individuals previously infected with SARS-CoV-1 were subsequently immunised with the SARS-CoV-2 BTN162b2 mRNA vaccine. Whether this can be achieved with a vaccination only approach remains to be determined. Another important consideration is that a vaccine that can drive production of NAb together with strong T cell responses which are less likely to be subverted by specific mutations, may provide broader and more durable protection against COVID-19, particularly against severe disease.

## Author Contributions

JT: manuscript concept, literature review, creation of figures, and writing and revising of manuscript; ME: manuscript revision and creation of figures; DG and TN: literature review and manuscript revision. All authors contributed to the article and approved the submitted version.

## Funding

This work was supported by a grant from NHMRC MRFF 2020 COVID-19 Vaccine Candidate Research, Australia (APP2013957). DG was supported by an NHMRC Investigator Award.

## Conflict of Interest

JT is an inventor of three provisional patents covering COVID-19 vaccines. DG is an inventor of two provisional patents covering COVID-19 vaccines and one covering a COVID-19 neutralising antibody test.

The remaining authors declare that the research was conducted in the absence of any commercial or financial relationships that could be construed as a potential conflict of interest.

## Publisher’s Note

All claims expressed in this article are solely those of the authors and do not necessarily represent those of their affiliated organizations, or those of the publisher, the editors and the reviewers. Any product that may be evaluated in this article, or claim that may be made by its manufacturer, is not guaranteed or endorsed by the publisher.
